# Gene Transcriptional and Metabolic Profile Changes in Mimetic Aging Mice Induced by D-Galactose

**DOI:** 10.1371/journal.pone.0132088

**Published:** 2015-07-15

**Authors:** Yue-Yue Zhou, Xiong-Fei Ji, Jian-Ping Fu, Xiao-Juan Zhu, Rong-Hua Li, Chang-Kao Mu, Chun-Lin Wang, Wei-Wei Song

**Affiliations:** 1 Key Laboratory of the Ministry of Education for Applied Marine Biotechnology, Ningbo University, Ningbo, China; 2 Collaborative Innovation Center for Zhejiang Marine High-efficiency and Healthy Aquaculture, Ningbo University, Ningbo, China; Institute of Oceanology, Chinese Academy of Sciences, CHINA

## Abstract

D-galactose injection has been shown to induce many changes in mice that represent accelerated aging. This mouse model has been widely used for pharmacological studies of anti-aging agents. The underlying mechanism of D-galactose induced aging remains unclear, however, it appears to relate to glucose and 1ipid metabolic disorders. Currently, there has yet to be a study that focuses on investigating gene expression changes in D-galactose aging mice. In this study, integrated analysis of gas chromatography/mass spectrometry-based metabonomics and gene expression profiles was used to investigate the changes in transcriptional and metabolic profiles in mimetic aging mice injected with D-galactose. Our findings demonstrated that 48 mRNAs were differentially expressed between control and D-galactose mice, and 51 potential biomarkers were identified at the metabolic level. The effects of D-galactose on aging could be attributed to glucose and 1ipid metabolic disorders, oxidative damage, accumulation of advanced glycation end products (AGEs), reduction in abnormal substance elimination, cell apoptosis, and insulin resistance.

## Introduction

Aging is an extremely complex and multifactorial process that exhibits universal, intrinsic, progressive, and deleterious characteristics[1]. According to the National Bureau of Statistics, people ≥60 years of age account for more than 14.9% of the population in China. In some developed countries, about 20% of the population are >60 years of age. With the expanding elderly population in the world, age-related diseases such as cardiovascular disease, cancer, and diabetes are becoming more common. Along with the pressing problem of age-related diseases, development of anti-aging agents has also become a popular research focus in recent years. Animal models of aging play an important role in anti-aging and drug discovery research. However, using natural aging models usually requires a long and unpredictable experimental time frame. Therefore, to facilitate anti-aging research, we need to create animal models that exhibit representative clinical symptoms of aging in response to designated experimental methods. Some of the current animal models of aging include D-galactose-induced mouse aging model, klotho mutant mouse, and SAMP strains of mice.

D-galactose-treated mice, created by Gong GQ in 1991[2], have been widely used in pharmacological studies of anti-aging agents[3–6]. This animal model exhibits many symptoms that resemble accelerated aging such as decreased activity of antioxidant enzymes, significant increase in malondialdehyde (MDA), accumulation of ROS, poor immune responses, and memory lapses[7–10]. This animal model has the advantages of fast response time and simple operation. Metabolic disorders, especially those involving glucose and lipid metabolism, have long been considered as a main factor contributing to the aging symptoms in D-galactose-treated mice[11]. Although changes in glucose and lipids are currently being investigated, the effects of D-galactose on other metabolites in mimetic aging mice have not received sufficient attention. Despite that the underlying mechanism of natural aging is still yet to be fully understood, it has been confirmed that aging is at least partially caused by changes in gene expression [12,13]. The programmed theories of aging, which argue that aging is naturally “programmed” into our genome, have been proposed to explain aging as a natural process. We, therefore, hypothesize that D-galactose can induce aging by affecting aging-related gene expression.

Metabonomics is a method used to study changes of endogenous small molecules in tissues and biological fluids caused by stimuli such as drugs, genetic effects, and disease processes[14–16]. Metabonomics has many advantages over conventional biochemical treatments. For instance, the metabonomic approach can sensitively monitor a large number of metabolites and can provide metabolic biomarkers that are useful for delineating groupings. Furthermore, metabonomics can measure the concentrations of the molecules of interest and identify their metabolite structures[17]. The use of powerful technologies like cDNA microarray allows us to measure expression levels of thousands of genes simultaneously. Since no study has been carried out to investigate the effect of D-galactose on gene expression, we propose that examining the RNA transcript profiles in D-galactose-treated mice can potentially reveal important changes in gene expression and genetic pathways that will shed light to the underlying mechanism of D-galactose induction of aging.

In this study, changes of metabolites in mimetic aging mice induced by D-galactose were studied comprehensively and systematically using the GC/MS-based metabonomics approach. Gene expression changes of D-galactose–treated mice were studied for the first time using the Agilent Mouse cDNA genechip.

## Materials and Methods

### Animals and treatments

D-galactose-induced mouse aging model was created as described previously[7]. Twenty male ICR mice (6 weeks of age, 21.32±3.34 g) were obtained from the Experimental Animal Center, Ningbo University, China. Mice were kept under constant temperature (24C±2°C) and humidity (60%) and were maintained on a reversed 12-h light: 12-h dark cycle. After acclimatization to the laboratory environment, ICR mice were randomly divided into two groups: control and D-galactose groups. The D-galactose group was injected with D-galactose (Sigma, St. Louis, MO) at a dose of 120 mg/kg/day for 6 weeks, while the control group was treated with saline (0.9%) of the same volume. Experimental verification of our D-galactose aging model showed that, as compared with the control group, the MDA content of D-galactose-treated mice was significantly increased. In addition, the activities of catalase (CAT), superoxide dismutase (SOD), and glutathione peroxidase(GSH-Px) were decreased significantly in D-galactose aging mouse livers and brains (S1 and S2 Tables). These observations validated our D-galactose aging mouse model. Liver, which is the central organ responsible for metabolism, plays a vital role in the synthetic decomposition and transformation of various bioactive metabolites. After a trial period of 45 days, the mice were euthanized by exsanguination under diethyl ether anesthesia. Before being euthanized, all mice were fasted 12 hours, but with ad libitum water. Mice were also protected from severe insults prior to euthanasia. Their livers ware removed immediately after exsanguination and stored at -80°C for later experiments. All experiments in this paper were conducted in compliance with the Chinese legislation regarding the use and care of laboratory animals and were approved by the Animal Care and Use Committee of Ningbo University (Permit Number: 0023472).

### GC-MS spectroscopy of mouse liver

Liver tissue samples (100 mg) were collected in 2 ml microcentrifuge tubes and 50 μl L-2-chlorophenylalanine (0.1 mg/ml stock in dH_2_O; Sigma) was added as an internal standard. This was followed by the addition of 0.5 ml of extraction liquid (Vmethanol:Vchloroform = 3:1) to homogenize the tissue in TissueLyser (Qiagen) for 5 min at 70 Hz. The sample was then centrifuged at 225g for 15 min at 4°C. Supernatant of 0.4 ml was transferred to a 2 ml glass autosampler vial, and allowed to dry in a vacuum concentration dryer without heating. Methoxyamination reagent (80 μl) (20 mg/ml in pyridine) was added to the glass autosampler vial at 37°C for 2 h with shaking. Then, BSTFA regent (0.1 ml) (1% TMCS, v/v) was added to the sample aliquot at 70°C for 1 h with shaking. GC/TOFMS analyses were performed when the temperatures dropped to room temperature.

Agilent 7890gas chromatograph system (Agilent 7890A, Agilent, USA) and Pegasus HT time-of-flight mass spectrometer (LECO Chroma TOF PEGASUS 4D, LECO, USA) were used for the GC/TOFMS analysis of samples. With sample injection set at splitless mode, a DB-5MS capillary column (30 m×250-μm inner diameter, 0.25-μm film thickness; J&W Scientific, Folsom,CA) was used for GC separation. The GC was operated at a flow rate of 20 ml min^-1^ using helium as the carrier gas and the injection temperature was set at 280°C. Temperature program was as follows: initial temperature at 50°C, holds for 1min, increases 10°C/min to 330°C, holds 5min. Electron ionization mass spectrometry at full scan mode (m/z 85–600) was used for MS analysis. The electron energy was set at -70 eV. The transfer line and ion source temperatures were 280°C and 220°C, respectively.

### Mouse liver mRNA profiles

Total RNA was extracted from the frozen liver using the mirVanaTM RNA Isolation Kit (Applied Biosystems, Darmstadt, Germany) and then cleaned with Qiagen RNeasyMini kit (Qiagen, Chatsworth, CA), according to the manufacturer’s instructions. The total RNA was quantified using NanoDrop ND-2000 (Thermo Scientific, Wilmington, DE) and the RNA integrity was evaluated using Agilent Bioanalyzer 2100 (Agilent Technologies, Palo Alto, CA). Next, double- strand cDNA was synthesized using PrimeScript RT reagent Kit (TaKaRa BIO, Shiga, Japan), and then labeled with cyanine-3-CTP. Microarray hybridization and the washing steps were performed according to the manufacturer's instructions. The arrays were scanned by Agilent Scanner G2505C in order to generate array image files.

### Microarray data validation by real-time polymerase chain reaction

Reverse transcriptase-polymerase chain reaction (RT-PCR) was performed to investigate the change in relative expression of genes (T-box 19, Cyp2r1, Jag1, Mfsd2a, Agk, Glo1). Assays were performed using the QuantiTect SYBR Green PCR kit (Qiagen, Hilden, Germany) in an Eppendorf Realplex Real-Time PCR system (Eppendorf, Hamburg, Germany). Gene expression levels were normalized to GAPDH. The experiments were performed in duplicate with liver samples prepared from 3 animals per group. The fold changes of the selected genes were analyzed by the 2^-ΔΔCT^ mothod [18].

### Data analysis

#### Statistical analysis of metabonomics

The primary data were imported into Micromass Markerlynx software version 4.1 (Waters Corporation, Milford, MA, USA) for preprocessing. The resulting three-dimensional data were analyzed by PCA (principal component analysis), PLS-DA (partial least squares discriminant analysis), and OPLS (orthogonal projections to latent structures-discriminant analysis) using SIMCA-P software Version 13.0 (Umetrics, Umeå, Sweden).

#### Statistical analysis of microarray data

Array image files were imported into Feature Extraction software, version 10.7.1.1(Agilent Technologies) for raw data extraction. Raw data were then normalized and analyzed by GeneSpring version 12.5 (AgilentTechnologies). The microarry probes with at least 1 out of 2 conditions flagged in “P” were chosen for further data analysis. Genes that were significantly differentially expressed between the two groups were identified as fold change ≥2.0 with P-value ≤0.05 (t-test).

## Results

### Results from metabonomics study

To determinate whether D-galactose influence metabolic pattern in control mice and to identify the metabolites that show the most significant change in concentrations, PCA,PLS-DA, and OPLS were used in the following GC/MS data analysis.

**Fig 1:**
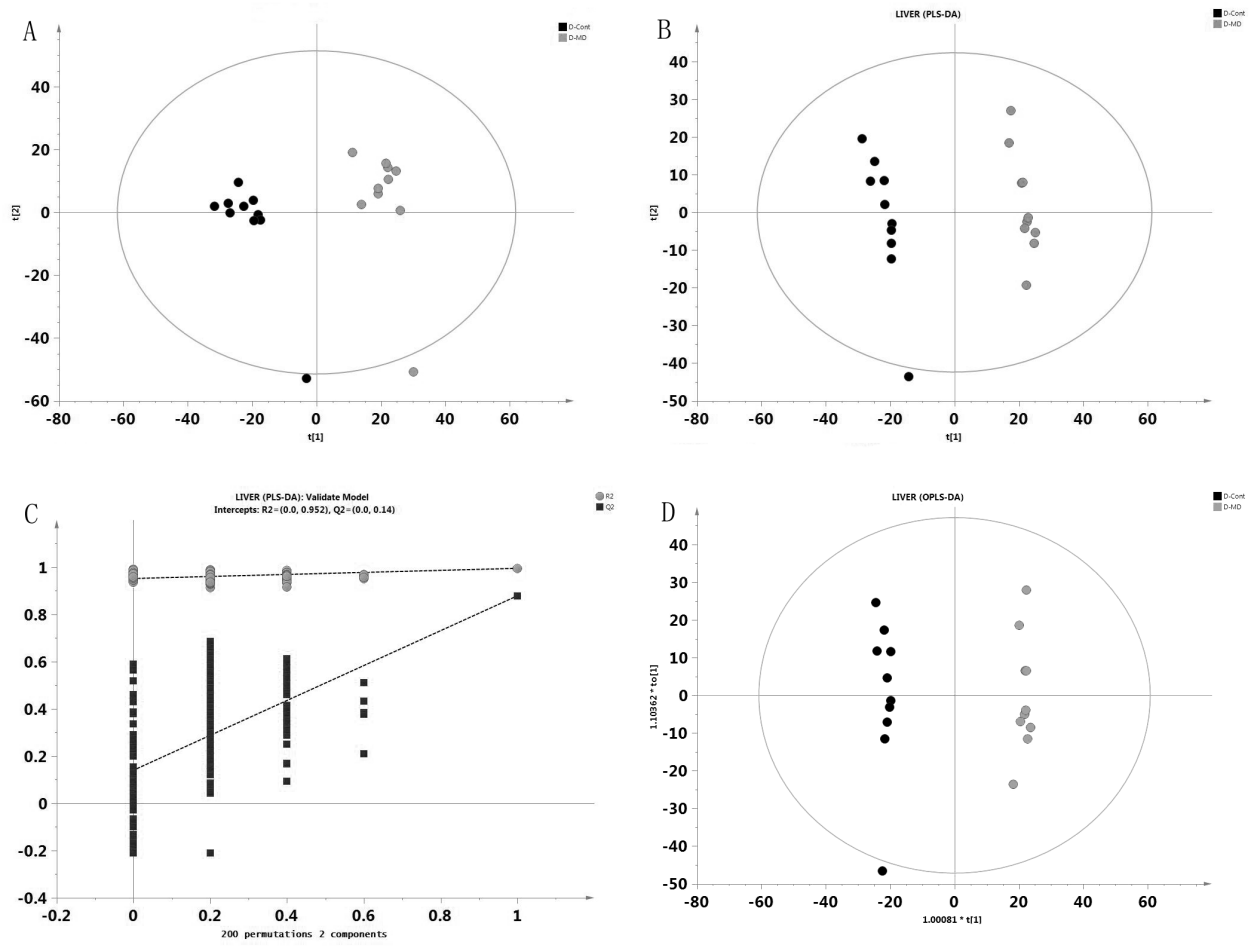
Multivariate statistical analysis of liver GC/MS data. A)Score plot of PCA obtained from control group(●) and D-galactose group(●). B)Score plot of PLS-DA obtained from control group(●) and D-galactose group(●). C) The result of permutation test, R2 (gray circle),Q2 (black square). D) Score plot of OPLS model obtained from control group(●) and D-galactose group(●)

Parameters collected from the control and the D-galactose-treated groups were clearly separated by PCA in 95% confidence intervals (Hotelling T2 ellipse) (Fig 1A). Results of PCA showed that the liver metabolic pattern in the D-galactose-treated group was significantly altered, indicating that D-galactose can induce serious metabolic changes in mouse liver. The PLS-DA model was constructed in order to widen the separation of the two groups, as well as a to better understand the variables that are responsible for clustering (Fig 1B). The results of PLS-DA showed that D-galactose treated group was clearly distinctive from the control group. The robustness and predictive ability of PLS-DA model were evaluated by Leave-one-out validation (LOOCV). R2Y = 0.995 and Q2Y = 0.879 (two parameters of LOOCV), which confirmed that the model was fit, stable and reliable for prediction. A permutation test was performed to further verify this model (Fig 1C). R2 and Q2 intercept values were (0.0,0.952) and (0.0,0.14), respectively. The low Q2 intercept value indicated the high reliability and high stability of this model. OPLS is a orthogonal rectification to PLS-DA model, OPLS—DA can filter out unrelated orthogonal signal, thus obtained different metabolites is more reliable(Fig 1D).

We screen significantly different metabolites through VIP(variable importance projection) of the first principal component in OPLS-DA model(VIP>1.0) and Student's T test(P<0.05). Metabolites which VIP>1 and p-value <0.05 were selected in Table 1. More details related to these metabolites can be found in S3 Table.

**Table 1:** Identification results of potential biomarkers detected by GC/MS in liver of D-galactose group. Metabolites which VIP>1 and p-value <0.05 have been showed in Table 1. If the value of log2 FOLD CHANGE is positive, the contect of that metabolite in D-galactose is greater than control group, If the value of log2 FOLD CHANGE is negative, the contect of that metabolite in D-galactose is less than control group.

Metabolite	RetentionTime	m/z	VIP	p-value	Fold change	log2 fold change
Fructose 1	17.4631	307	1.98432	0.03276	3.156158	1.658169
Sophorose 2	25.2294	319	4.33718	0.003095	7534780	22.84513
Palmitoleic acid	19.026	311	3.62036	0.033467	0.287164	-1.80005
Isoleucine	9.00167	86	2.15906	0.04119	3.560789	1.832197
Lactic acid	7.26854	219	4.25708	4.18E-06	8.537517	3.093817
Arachidonic acid	22.1227	108	2.10906	0.002832	3.083468	1.624554
Xanthine	18.9413	243	1.38454	0.019336	0.599061	-0.73923
D-Talose 2	17.9603	211	1.62746	0.04413	0.408442	-1.2918
Sorbitol	18.1324	244	2.43229	0.002016	220.8209	7.786733
Glutamine 3	13.7833	155	3.87248	0.029609	0.437491	-1.19267
Oxoproline	13.6611	336	1.59867	0.016554	2.356776	1.236814
Phosphate	10.3103	158	5.66992	1.05E-11	4.86E+08	28.85675
Xylose 1	15.1943	103	4.05294	4.17E-07	17.33121	4.1153
Fucose 1	15.946	160	1.96502	0.019886	5.896048	2.559748
Xylose 2	15.0501	217	1.91482	0.002226	3.49752	1.806332
Cellobiose 1	24.4233	361	1.46246	0.0069	14.94889	3.901967
Erythrose 2	12.49	201	2.86674	0.015468	1856007	20.82377
Fructose-6-phosphate	21.3829	156	2.37331	0.011185	0.126768	-2.97974
1,5-Anhydroglucitol	17.1617	217	2.07223	0.02223	0.170882	-2.54893
D-galacturonic acid 2	18.2821	299	1.32656	0.006003	1.985	0.989139
Oxamic acid	10.2096	171	3.98259	0.000112	3.616163	1.85446
Inosine	23.6621	93	3.16985	0.010008	7748968	22.88557
Cholesterol	27.9683	211	3.19993	0.001297	9.494937	3.247158
Uric acid	19.6589	398	2.00843	0.004495	0.223693	-2.16041
D-erythro-sphingosine 1	22.7864	387	3.25372	1.44E-05	525083.8	19.00219
Adenosine-5-monophosphate	27.0061	257	2.18523	0.004598	0.224391	-2.15591
1-Methyladenosine 2	22.0599	361	1.15883	0.028768	0.019626	-5.67107
Allantoic acid 2	17.5249	156	2.17308	0.011752	4E-07	-21.2539
Uridine 2	22.6408	173	2.60916	0.002197	2.43E-06	-18.6519
Oxalic acid	8.02167	114	2.41541	0.001542	10.88661	3.444483
Glucose-1-phosphate	16.4466	292	2.08992	0.005905	8.182085	3.032468
2-Deoxy-D-galactose 1	16.5897	217	1.31075	0.037993	0.393344	-1.34614
Elaidic acid	20.84	253	1.62197	0.001097	3.369196	1.752404
Hypoxanthine 2	19.1147	325	2.01796	0.000235	0.232314	-2.10585
Alanine 2	11.6406	102	2.39355	0.020381	4.294856	2.10261
Spermidine 1	21.2628	269	2.07336	0.011037	0.177932	-2.4906

Table 1 showed that glycometabolism, fatty acid metabolism, transamination, purine metabolites, and some other important metabolites were disturbed in the D-galactose aging group, which may play an vital important role in aging programs.

#### D-galactose results in metabolic disorders of carbohydrates in the liver

Compared with control group, liver samples from the D-galactose group contained more than eight times the amount of glucose-1-phosphate. In addition, with the exception of D-Talose-2, the levels of fructose-1, sophorose-2, xylose-1, fucose-1, xylose-2, cellobiose-1, and erythrose-2 were also higher in the D-galactose group. Large amounts of lactic acid, which is the end product of glycolysis, along with decreased levels of fructose-6-phosphate, were detected in our D-galactose group, which was likely the result of increased glycolysis driven by the excessive amount of sugar in the mouse liver.

#### Disturbance in lipid metabolism was detected in D-galactose mice

A significant increase in cholesterol concentration was discovered in the D-galactose mouse liver, which was similar to previous reports [11]. Cholesterol is a precursor of steroid hormones and an essential membrane component. Furthermore, cholesterol has been shown to play crucial roles in certain age-related diseases, such as Alzheimer’s diseases, cardiovascular disease, and non-alcoholic fatty liver disease [19,20]. Aging has been shown to increase concentrations of cholesterol precursors (lanosterol and lathosterol) in the liver [21]. In D-galactose mouse livers, we observed a signficant increase in concentration of elaidic acid, which can exert atherogenic effects and lead to cardiovascular disease due to its trans configuration [22]. In addition, palmitoleic acid content was significantly reduced in D-galactose mice. Palmitoleic acid has been shown to improve hepatic lipid metabolism. It can also attenuate hyperglycemia and hypertriglyceridemia by increasing insulin sensitivity [23]. The level of arachidonic acid was also significantly inscreased in D-galactose mouse liver. Arachidonic acid, a polyunsaturated fatty acid, serves as an important component of membrane lipids.

#### Transamination was enhanced in D-galactose mice

Transamination is a key step in the biosynthesis of most amino acids. Glutamine and alanine is the reaction substrate and product of transamination respectively. A rapid increase in alanine and a decrease in glutamine were observed in the D-galactose group, which might be attributed to the transamination of excessive glucose. Glutamine is a rate-limiting substrate in GSH (glutathione) formation. Glutamine and cysteine can form GC (γ-glutamylcysteine) in an ATP-dependent reaction. Upon addition of glycine, GC can form GSH. GSH, a quencher for free radicals and a conjugate that enhances drug solubility in water, declines in both natural aging and D-galactose aging mouse liver [24].

#### Purine metabolic disorder was detected in D-galactose aging mice

Adenosine 5-monophosphate, l-methyladenosine, xanthine, hypoxanthine, uric acid, allantoic acid, and inosine are intermediates of purine metabolism. We observed that the level of most of these molecules were reduced in D-galactose mice, except for inosine. It has reported that the contect of inosine increased in senescent C57/Bl6 mice[25]. The net release of inosine was enhanced by in vitro aging of lung fibroblasts and had been shown to increase in skin fibroblasts from aged donors[26]. These studies is consistent with our findings. However, there is no more researches on the potential relationship between aging and purine metabolism in mouse liver.

#### Abnormal levels of metabolic molecules were observed in livers of D-galactose mice

The levels of sorbitol, sphingosine, and phosphate were increased in D-galactose mouse liver. Sorbitol is a slow-metabolizing isomer of galactitol, which tends to accumulate and causes harmful effects on the body. The levels of sorbitol were found to increase with aging in lens and sciatic nerves [27]. Sphingosine, an 18-carbon amino alcohol, plays important roles in signal transduction during cell apoptosis [27,28]. It has been shown that the basal levels of sphingosine increases in naturally aging animals, which may contribute to the onset of inflammatory like symptoms in aging livers [29]. Phosphate is a vital component of phospholipids in cell membranes and is involved in intermediate cellular signaling [30]. Excess amounts of phosphorus were found in klotho mutant mice [31]. Spermidine participates in multiple biological processes including autophagy induction, which is important in protecting cells from various noxious agents and extending cell lifespan. Spermidine has also been shown to significantly alleviate age-related oxidative protein damages in mice. During hyperglycemia, concentration of 1,5-Anhydroglucito can be dramatically reduced due to impaired tubular reabsorption in the kidneys and thus, level of 1,5-Anhydroglucitol can be used to assess glycemic variability in diabetic patients [32]. The levels of spermidine and 1,5-anhydroglucitol were both decreased in our aging mouse livers, which could potentially explain some of the harmful effects of aging seen in our mouse model.

### Results from Microarray study

Compared with control mice, mRNA expression of 30 and 18 genes were down and up regulated in D-galactose-induced aging mice, respectively (absolute fold-change ≥2; P-value ≤0.05). Table 2 listed the parts of genes that showed significantly different expression. More details related to the mRNA expression are included in S4 Table.

**Table 2:** Significant mRNA changes induced in liver of D-galactose group.

Symbol	Description	ProbeName	p-value	Fold change	Regulation
Tbx19	Mus musculus T-box 19 (Tbx19)	A_52_P95930	4.54E-04	9.761589	up
Hc	Mus musculus hemolytic complement (Hc)	A_51_P155323	0.047217	2.608894	up
Lepr	Mus musculus leptin receptor (Lepr), transcript variant 2	A_55_P2177911	0.027683	2.059761	up
Bhmt	Mus musculus betaine-homocysteine methyltransferase (Bhmt)	A_55_P2105180	0.0267	5.115597	up
Klk1b26	Mus musculus kallikrein 1-related petidase b26 (Klk1b26)	A_55_P2001474	0.001978	2.81843	down
Olfr1211	Mus musculus olfactory receptor 1211 (Olfr1211)	A_51_P237106	0.0418	2.02142	up
Dusp6	Mus musculus dual specificity phosphatase 6 (Dusp6)	A_51_P502614	0.028695	2.087547	up
Olfr1408	Mus musculus olfactory receptor 1408 (Olfr1408)	A_55_P2164683	0.005581	2.530703	down
Ube2l6	Mus musculus ubiquitin-conjugating enzyme E2L 6 (Ube2l6)	A_55_P2031125	0.036739	2.236952	down
Ascl4	Mus musculus achaete-scute complex homolog 4 (Drosophila) (Ascl4)	A_55_P1993148	0.025012	5.178002	up
Bcl6	Mus musculus B cell leukemia/lymphoma 6 (Bcl6)	A_52_P161495	2.16E-04	2.146329	up
Bivm	Mus musculus basic, immunoglobulin-like variable motif containing (Bivm)	A_55_P2170109	0.011495	2.046546	up
Cyp2r1	Mus musculus cytochrome P450, family 2, subfamily r, polypeptide 1 (Cyp2r1)	A_55_P1966755	0.041499	2.40151	down
Jag1	Mus musculus jagged 1 (Jag1)	A_52_P634090	0.04412	2.077494	down
Atp6v0d2	Mus musculus ATPase, H+ transporting, lysosomal V0 subunit D2 (Atp6v0d2)	A_66_P124179	0.025687	2.154945	up
Mfsd2a	Mus musculus major facilitator superfamily domain containing 2A (Mfsd2a)	A_51_P279437	0.017035	2.116091	down
Bhmt	Mus musculus betaine-homocysteine methyltransferase (Bhmt)	A_55_P2105181	0.033883	4.284998	up
Agk	Mus musculus acylglycerol kinase (Agk), nuclear gene encoding mitochondrial protein	A_55_P2063163	0.005518	3.043666	up
Tmtc3	Mus musculus transmembrane and tetratricopeptide repeat containing 3 (Tmtc3), transcript variant 2	A_51_P212473	0.005088	2.04044	up
Glo1	Mus musculus glyoxalase 1 (Glo1), transcript variant 1	A_51_P480982	0.049039	2.045152	down

#### Microarray results were validated by RT-PCR

RT-PCR was performed on six genes (Bhmt-F,T-box 19, Cyp2r1, Jag1, Mfsd2a, Agk, Glo1) in order to independently verify some of our microarray results (Table 3). Consistent with our microarray expression profiling data, expressions of Bhmt-F, T-box 19, Agk were found to be upregulated in the D-galactose groups (Table 3). However, expressions of Cyp2r1, Jag1, Mfsd2a and Glo1 were downregulated in D-galactose groups. The RT-PCR results corresponded to that of the cDNA microarray, with a strong correlation (r^2^ = 0.995, P <0.01) between the fold change values generated by the cDNA microarray and the RT-PCR analyses.

**Table 3:** Independent validation by real-time PCR of 6 genes significantly altered in the liver of D-galactose mice.

Gene	RT-PCR		Affymetrix	
	Fold change	P value	Fold change	P value
betaine-homocysteine methyltransferase (Bhmt)	3.588	<0.05	5.116	<0.05
T-box 19 (Tbx19)	7.007	<0.05	9.762	<0.01
cytochrome P450, family 2, subfamily r, polypeptide 1 (Cyp2r1)	2.271	<0.05	2.402	<0.05
jagged 1 (Jag1)	1.835	<0.05	2.077	<0.05
major facilitator superfamily domain containing 2A (Mfsd2a)	2.026	<0.05	2.116	<0.05
acylglycerol kinase (Agk)	2.815	<0.01	3.044	<0.05
glyoxalase 1 (Glo1)	2.075	<0.01	2.045	<0.05

#### D-galactose might affect cell apoptosis and progression to vascular diseases in mice

D-galactose induces changes in expression of genes associated with cell apoptosis and vascular diseases (Dusp6, Egr-1, mJagged1, KLK1b26). Dephosphorylating the critical phosphothreonine and phosphotyrosine residues within extracellular signal-regulated kinase (ERK) is the function of Dusp6. ERK is an important signal transduction enzyme that mediates cell proliferation and programmed cell death. The expression of Dusp6 can induce cell apoptosis by inactivating ERK and inhibiting cell proliferation [33]. The level of Egr1 was increased in our D-galactose aging mice, Egr1 can stimulate apoptosis by transactivating the p53 gene[34] and decrease insulin sensitivity [35]. mJagged1 is directly associated with multiple Notch receptors and is involved in the mediating Notch signaling [36–38]. The decrease mJagged1 can promote proliferation of vascular smooth muscle cells (VSMCs) through Notch signaling pathway[39] and then lead to vascular diseases. We observed a decrease in Jagged1 expression in our D-galactose mice which could potentially promote proliferation of vascular smooth muscle cells (VSMCs) through Notch signaling pathway. Kallikrein 1-related petidase b26 (KLK1b26), a member of the kallikrein 1-related petidases (KLK1), can inhibit proliferation of VSMCs [40]. The expression of KLK1b26 was reduced in our D-galactose mice, which could further promote proliferation of VSMCs. Excessive VSMCs is a common pathological feature of the development and progression of atherosclerosis and hypertension [40,41].

#### D-galactose might lead to impaired degradation mechanisms in mice

The mRNA expression levels of genes involved in the elimination of abnormal substances (Ube2l6, Cyp2r1,GLO1) were downregulated in D-galactose mice, which could potentially be responsible for some of the aging symptoms of our aging mice.Ubiquitin-conjugating enzyme E2L6 (Ube2l6) is a component of the ubiquitin system that enable the degradation of short-lived and abnormal proteins[42]. Conjugating activity and proteolytic capability of ubiquitin can be inhibited by oxidative damage, which ultimately contribute to the accumulation of deformed proteins in oxidatively challenged aging lens [43]. CYP2R1 (Cytochrome P450, family 2, subfamily r, and polypeptide 1) belongs to the cytochrome P450 family,which plays an important role in detoxification, drug metabolism, and removing waste products [44]. The enzyme GLO1 is an integral component of the glyoxalase system. A major function of the glyoxalase pathway is believed to be detoxification of α-ketoaldehydes, especially the cytotoxic metabolite methylglyoxal (MG) [45].

#### D-galactose injection might affect immune responses in aging mouse model

The mRNA expression levels of immune-system-regulating genes (Hc, BIVM and Tmtc3) were upregulated in our D-galactose mice. Hemolytic complement (Hc,C5) belongs to the complement system, which plays key roles in immunity and inflammation. Hc can induce histamine release from basophilic leukocytes as well as from mast cells and stimulate polymorphonuclear leukocytes migration directly to the site of inflammation. In addition, excessive accumulation of complement proteins is the major cause of drusens, whose deposition in the eye is an early sign of age-related macular degeration (AMD) [46]. BIVM possesses Ig-type motifs by including short peptide motifs characteristic of an immunoglobulin (Ig) variable (V) region[47]. Tmtc3 have an impact on the function of immune cell by modulating XBP-1 transcript expression and proteasome activity. [48]. However, there are no studys about the relationship between aging and BIVM and Tmtc3.

#### D-galactose might affect development in aging mice

The transcription levels of of development-related genes (Tbx19, ASCL4, CaMK-II) were also increased significantly in D-galactose mice. Tbx19 (T-box19) encodes transcription factors that are involved in developmental regulation. ASCL4 is a transcription factor important in cell differentiation and cell fate determination [49]. CaMK-II encodes a multifunctional Ca2/calmodulin-dependent protein kinase,which plays crucial roles during early development and gametogenesis [50]. There are no studys about the relationship between aging and Tbx19, ASCL4, CaMK-II, but aging is caused by regulating development (such as growth hormone) that has been proved[51].

#### D-galactose also affects expression of other genes

The mRNA expression of olfactory receptor gene Olfr1408 and Mfsd2a were descreased in D-galactose mice. The ability of rodents to detect and discriminate odors declines with age. Although the underlying cellular and molecular mechanism for this phenomenon is largely unknown, previous report has demonstrated that expression of olfactory receptor gene varies significantly during the normal aging process [52]. Mfsd2a encodes the important carrier protein for docosahexaenoic acid (DHA). Brain DHA content has been shown to decrease significantly in *Mfsd2a*-deficient (*Mfsd2a*-knockout) mice and its deficiency could lead to cognitive deficits and severe anxiety [53]. In addition, we also observed increase in LEP-R and AGK expression in D-galactose mice. LEP-R, a single transmembrane domain receptor of the cytokinereceptor family, functions as a receptor for leptin, which is an adipocyte-derived hormone that activates the sympathetic nervous system, increases energy expenditure, and decreases food intake [54]. Acylglycerol kinase (AGK) is a lipid kinase that phosphorylates either monoacylglycerol to form lysophosphatidic acid (LPA) or phosphorylate diacylglycerol to form phosphatidic acid (PA) [55]. LPA, a multifunctional phospholipid mediator, is involved in a wide range of pathological processes such as hypertension and tumor cell invasion [56]. In addition, AGK expression has also been reported to be upregulated in cancer [57].

## Discussion

### Oxidative damage and sorbtiol toxicity can lead to aging symptoms observed in D-galactose mice

There are three different metabolic pathways for galactose in the body. The most important pathway is the Leloir pathway, in which galactose is rapidly metabolized into glucose-1-phosphate, a substrate in glycolysis, by four consecutive enzymes. In another pathway, galactose is converted into galacitol (ducitol) by aldose reductase. In the third pathway, galactose-oxidase acts on ducitol and O_2_ to produce aldehydes and H_2_O_2_ [58]. Many studies have demonstrated the potential oxidative damage by H_2_O_2_ and O_2_^-^, which could accumulated from excessive galactose metabolism, is an important cause of aging in D-galactose mice. It has also been reported that superoxide content increased dramatically in brains and livers of D-galactose aging mice as demonstrated by electron spin resonance spectroscopy (ESR) [59]. Verification of our D-galactose aging model shows that, as compared with control group, the MDA content of D-galactose-treated mice increased significantly while the activities of CAT, SOD, and GSH-Px decreased significantly. These results are in agreement with published experimental data [7] and indicate the validity of our D-galactose model. These results also support the hypothesis that D-galactose-induced aging symptoms are caused by oxidative stress[60].

Galacitol is also produced by excessive metabolism of galactose. We hypothesized that the tissue toxicity of galacitol could be another cause of aging in our D-galactose mice. We observed that sorbitol, the isomer of galactitol, was increased significantly in D-galactose treated mouse livers. Sorbtiol is similar to galacittol in its physiological functions. The accumulation of Sorbitol is known to be harmful to the body[61]. Besides, in certain aging organs such as the lens and the sciatic nerve, the levels of sorbitol were increased significantly after D-galactose injection. Although the relationship between increased galacitol level and aging in D-galactose mice has not yet been established, our study suggests that sorbitol, an isomer of galactitol, might play an important role in the aging process of D-galactose mice.

### The process of converting excess glucose into fat and proteins could potentially lead to cardiovascular diseases in D-galactose mice

The level of glucose-1-phosphate was increased significantly in our D-galactose mice. This could promote the process of converting glucose into fat and proteins. In fact, a significant increase in cholesterol, elaidic acid, arachidonic acid concentrations were discovered in our D-galactose mouse liver. In addition, a rapid increase of alanine and decrease of glutamine were observed in the D-galactose mice. All of these results indicate that high concentration of glucose could promote conversion of glucose to fat and transamination. High fat content increases the risk of age-related diseases, especially cardiovascular disease. Furthermore, cholesterol plays crucial roles in Alzheimer’s diseases and non-alcoholic fatty liver disease. Elaidic acid, in particular, possesses atherogenic effects and could lead to cardiovascular diseases [21,22]. Dikran et al. also reported that GSH, a quencher for free radicals and a drug conjugant, declined in naturally aging mouse liver and in D-galactose mouse liver [24]. We speculated that transamination leads to a rapid decrease of glutamine, which in turn, allows glutamine to become a rate-limiting substrate for GSH (glutathione) formation and ultimately leads to the decrease in GSH level. The decreased Jagged1 expression was also observed in D-galactose mice. The Jagged1 protein mediates Notch signaling and its potential effect on vascular diseases by associating directly with multiple Notch receptors [36,37]. In D-galactose mice, the decrease in mJagged1 could promote the proliferation of vascular smooth muscle cells (VSMCs) through the Notch signaling pathway [39]. On the other hand, kallikrein 1-related petidase b26(KLK1b26), a member of the kallikrein 1-related petidases (KLK1), has the opposite effect on the proliferation of VSMCs. In fact, we observed a decrease in KLK1b26 expression in our D-galactose mice, which could further promote the proliferation of VSMCs. Excessive proliferation of VSMCs is a common pathological feature of atherosclerosis and hypertension, both of which become more common with age.

### The levels of advanced glycation end products (AGEs) were increased in D-galactose mouse livers

The levels of reducing sugars (fructose1, sophorose2, xylose1, fucose1, xylose2, and erythrose 2) were significantly increased in D-galactose mice. The high concentration of reducing sugars could lead to an increase in Maillard reaction and the accumulation of AGE, which coule be harmful and could contribute to aging by crosslinking proteins and inducing inflammatory responses [62]. Previously studies have demonstrated that AGE level is significantly increased in D-galactose aging mice [63].

### Impaired elimination of abnormal substances could contribute to aging in D-galactose mice

Spermidine induces autophagy, which in turn, mediates cytoprotection against a variety of noxious agents and thereby promotes longevity [64,65]. The level of spermidine was decreased in D-galactose mouse liver, which suggests the impaired elimination of foreign substances in the D-galactose induced aging mice. Ubiquitin-conjugating enzyme E2L 6 (Ube2l6) is a component of the ubiquitin system that is responsible for targeting abnormal proteins for degradation. In fact, the level of Ube2l6 was also decreased in D-galactose mouse livers, further indicating a potential deficiency in normal housekeeping functions in D-galactose mice. Reactive oxygen species such as H_2_O_2_ and O_2_^-^, which are produced by excessive galactose metabolism, could also explain some of the harmful effect of the aging process, mostly due to the observation that oxidative stress can inactivate the ubiquitin conjugation activity by suppressing proteolytic capability [43]. The level of Cytochrome P450 was also decreased in our D-galactose aging mice. Cytochrome P450 is responsible for the biotransformation of most foreign substances in the liver, including 70–80% of all drugs used in the clinical setting [44]. Furthermore, we also observed a decrease in GLO1 level in the D-galactose mice. The GLO1 enzyme is an integral component of the glyoxalase system. A major function of the glyoxalase pathway is believed to be the detoxification of α-ketoaldehydes, particularly a cytotoxic metabolite methylglyoxal (MG) [45].

### Accelerated apoptosis and enhanced insulin resistance oberseved in D-galactose mice

Dusp6 has been shown to selectively dephosphorylate the critical phosphothreonine and phosphotyrosine residues within the extracellular signal-regulated kinase (ERK), which is an important signal transducing enzyme involved in mediating cell proliferation and programmed cell death. The decrease of Dusp6 expression level can induce cell apoptosis by inactivating ERK and thereby, inhibit cell proliferation [33]. The level of Dusp6 was decreased in our D-galactose aging mice, which could explain the accelerated cell apoptosis commonly observed in aging.

The level of Egr1 was increased in our D-galactose aging mice, which could be the result of increased oxidative stress and glucose level. It has been demonstrated that oxidative stress can activate the MAPK signaling pathways [66,67]. MAPK, in turn, can upregulate the expression of Egr1. EGR1 stimulates apoptosis by transactivating the p53 gene [34]. High glucose level can also lead to an increase in Egr1 expression. EGR1 has been shown to promote insulin resistance [35]. Furthermore, the palmitoleic acid content was significantly reduced in our D-galactose mouse liver. Palmitoleic acid has been reported to improve hepatic lipid metabolism and attenuate hyperglycemia and hypertriglyceridemia by increasing insulin sensitivity [23]. Many studies have also supported our results by demonstrating that hyperglycemia, hyperinsulinemia, insulin resistance, and hyperlipidemia often occur in D-galactose induced mice [68,69], which may be consequences of the high level of Egr1 expression and the decrease in palmitoleic acid in these mice. Insulin resistance is a primary cause of age-related diseases such as type 2 diabetes, atherosclerosis, and nonalcoholic fatty liver disease (NAFLD) [70].

## Conclusion

Metabonomic analysis based on GC/MS and gene expression profile generated by Agilent Mouse mRNA gene chip was performed in this study to investigate changes in the livers of D-galactose induced mice. Based on results of PCA and PLS-DA, parameters collected from the D-galactose treated group and the control group can be clearly distinguished from one another. Using an OPLS model, 51 potential biomarkers were identified in our D-galactose mice at the metabolic level. Forty-nine mRNAs were differentially expressed between control and D-galactose mice. Integrated analysis of the data generated from metabonomics and transcriptomics show that the mechanisms behind D-galactose induction aging likely involve glucose and 1ipid metabolic disorders, increased oxidative damage, accumulation of advanced glycation end products (AGEs), impaired elimination of abnormal substances, accelerated cell apoptosis, and enhanced insulin resistance. The D-galactose mouse model has been widely used for pharmacological studies of anti-aging agents. Our study further confirms the validity of this model by providing an explanation for the possible mechanisms of D-galactose aging induction through promoting metabolic disorders and oxidative damage. Our study is also the first of its kind that suggests D-galactose could induce aging by altering the gene expressions in mice, which would encourage and facilitate future research on development of more effective anti-aging agents.

## Supporting Information

S1 Table The effect of D-galactose on MDA content and the activities of CAT, SOD, GSH-Px in mice liver. Note: # p<0.05, ## p<0.01 versus normal group
(DOCX)

S2 Table The effect of D-galactose on MDA content and the activities of CAT, SOD, GSH-Px in mice brain. Note: # p<0.05, ## p<0.01 versus normal group.
(DOCX)

S3 Table Details of potential biomarkers detected by GC/MS in liver of D-galactose group. (XLSX)

S4 Table Details of significant mRNA changes induced in liver of D-galactose group. (DOCX)

S5 Table The results of total RNA extraction. (DOCX)

S6 Table Standardize data of mRNA chip. (XLSX)

S7 Table The technology roadmap of our experiment and the explanation of supporting information. (DOCX)
