# Supplementary material for: Gene Transcriptional and Metabolic Profile Changes in Mimetic Aging Mice Induced by D-Galactose
Source: PLoS One. 2015 Jul 15;10(7):e0132088. doi: 10.1371/journal.pone.0132088 (PMC4503422; doi:10.1371/journal.pone.0132088)
Supplement: S1 Table — Note: # p<0.05, ## p<0.01 versus normal group (DOCX) [file pone.0132088.s001.docx]

**S1 Table . The effect of D-galactose on MDA content and the activities of CAT，SOD，GSH－Px in mice liver**

| Group | Does  (mg·kg^-1^·d^-1^) | MDA  （nmoL/mg·pro） | CAT  （U/mL） | SOD  （U/mL） | GSH-Px  （U/mL） |
| --- | --- | --- | --- | --- | --- |
| Control | - | 2.28±0.48 | 209.49±36.33 | 258.75±33.32 | 979.65±93.02 |
| D-galactose | - | 3.89±0.43## | 151.85±12.77# | 198.48±13.07## | 686.13±91.83## |

Note: # p<0.05，## p<0.01 versus normal group
