# Supplementary material for: Gene Transcriptional and Metabolic Profile Changes in Mimetic Aging Mice Induced by D-Galactose
Source: PLoS One. 2015 Jul 15;10(7):e0132088. doi: 10.1371/journal.pone.0132088 (PMC4503422; doi:10.1371/journal.pone.0132088)
Supplement: S2 Table — Note: # p<0.05, ## p<0.01 versus normal group. (DOCX) [file pone.0132088.s002.docx]

**S2 Table .The effect of D-galactose on MDA content and the activities of CAT，SOD，GSH－Px in mice brain**

| Group | Does  (mg·kg^-1^·d^-1^) | MDA  （nmoL/mg·pro） | CAT  （U/mL） | SOD  （U/mL） | GSH-Px  （U/mL） |
| --- | --- | --- | --- | --- | --- |
| Control | - | 2.89±0.63 | 2.40±0.37 | 116.84±11.80 | 65.52±6.95 |
| D-galactose | - | 4.12±0.93## | 1.17±0.43# | 96.00±13.00# | 48.42±9.25# |

Note: # p<0.05，## p<0.01 versus normal group
