# Supplementary material for: Gene Transcriptional and Metabolic Profile Changes in Mimetic Aging Mice Induced by D-Galactose
Source: PLoS One. 2015 Jul 15;10(7):e0132088. doi: 10.1371/journal.pone.0132088 (PMC4503422; doi:10.1371/journal.pone.0132088)
Supplement: S4 Table — (DOCX) [file pone.0132088.s004.docx]

| Symbol | Description | ProbeName | p | FOLD CHANGE | Regulation |
| --- | --- | --- | --- | --- | --- |
| Csn3 | casein kappa [Source:MGI Symbol;Acc:MGI:107461] [ENSMUST00000001667] | A_51_P227275 | 0.005618 | 2.2435272 | down |
| Tbx19 | Mus musculus T-box 19 (Tbx19), mRNA [NM_032005] | A_52_P95930 | 4.54E-04 | 9.761589 | up |
| Hc | Mus musculus hemolytic complement (Hc), mRNA [NM_010406] | A_51_P155323 | 0.047217 | 2.6088936 | up |
| Lepr | Mus musculus leptin receptor (Lepr), transcript variant 2, mRNA [NM_010704] | A_55_P2177911 | 0.027683 | 2.0597606 | up |
| Bhmt | Mus musculus betaine-homocysteine methyltransferase (Bhmt), mRNA [NM_016668] | A_55_P2105180 | 0.0267 | 5.1155972 | up |
| Klk1b26 | Mus musculus kallikrein 1-related petidase b26 (Klk1b26), mRNA [NM_010644] | A_55_P2001474 | 0.001978 | 2.8184302 | down |
| Olfr1211 | Mus musculus olfactory receptor 1211 (Olfr1211), mRNA [NM_001011804] | A_51_P237106 | 0.0418 | 2.0214198 | up |
| C330020G15Rik | Mus musculus ES cells cDNA, RIKEN full-length enriched library, clone:C330020G15 product:unclassifiable, full insert sequence. [AK082796] | A_55_P2367007 | 0.007953 | 3.7465405 | down |
| Hist1h1c | Mus musculus histone cluster 1, H1c (Hist1h1c), mRNA [NM_015786] | A_51_P516133 | 0.038989 | 2.9715626 | down |
| Gm5463 | Mus musculus 13 days embryo heart cDNA, RIKEN full-length enriched library, clone:D330001M17 product:unclassifiable, full insert sequence. [AK052160] | A_55_P2091801 | 0.033448 | 2.4108589 | up |
| Egr1 | Mus musculus early growth response 1 (Egr1), mRNA [NM_007913] | A_51_P367866 | 0.034083 | 2.79274 | up |
|  |  | A_55_P2120389 | 0.020034 | 2.1565766 | up |
| Dusp6 | Mus musculus dual specificity phosphatase 6 (Dusp6), mRNA [NM_026268] | A_51_P502614 | 0.028695 | 2.0875468 | up |
| Gm3448 | Mus musculus predicted gene 3448 (Gm3448), mRNA [NM_001123367] | A_55_P1987196 | 0.046641 | 2.1094868 | down |
| A830052D11Rik | Mus musculus RIKEN cDNA A830052D11 gene (A830052D11Rik), non-coding RNA [NR_045403] | A_55_P2412319 | 0.039322 | 2.440551 | up |
| Olfr1408 | Mus musculus olfactory receptor 1408 (Olfr1408), mRNA [NM_146764] | A_55_P2164683 | 0.005581 | 2.5307033 | down |
| Bbs7 | Mus musculus Bardet-Biedl syndrome 7 (human) (Bbs7), mRNA [NM_027810] | A_55_P2005868 | 0.020595 | 2.2286725 | down |
| Gm3142 | PREDICTED: Mus musculus predicted gene 3142 (Gm3142), mRNA [XM_001475809] | A_55_P1996746 | 0.018043 | 2.2501893 | up |
| Ube2l6 | Mus musculus ubiquitin-conjugating enzyme E2L 6 (Ube2l6), mRNA [NM_019949] | A_55_P2031125 | 0.036739 | 2.236952 | down |
| Ascl4 | Mus musculus achaete-scute complex homolog 4 (Drosophila) (Ascl4), mRNA [NM_001163614] | A_55_P1993148 | 0.025012 | 5.178002 | up |
| Gm10030 | Mus musculus 0 day neonate lung cDNA, RIKEN full-length enriched library, clone:E030015K02 product:hypothetical protein, full insert sequence. [AK086953] | A_52_P548202 | 0.003792 | 4.7964244 | down |
| Bcl6 | Mus musculus B cell leukemia/lymphoma 6 (Bcl6), mRNA [NM_009744] | A_52_P161495 | 2.16E-04 | 2.1463287 | up |
| Gm5589 | Mus musculus predicted gene, 434166, mRNA (cDNA clone IMAGE:6493658). [BC080727] | A_55_P2078830 | 0.004596 | 2.5126987 | down |
| Gm15234 | Mus musculus 0 day neonate thymus cDNA, RIKEN full-length enriched library, clone:A430006A04 product:unclassifiable, full insert sequence. [AK039813] | A_66_P103260 | 0.015709 | 2.170392 | up |
| 5730414N17Rik | Mus musculus 8 days embryo whole body cDNA, RIKEN full-length enriched library, clone:5730414N17 product:unclassifiable, full insert sequence. [AK017561] | A_55_P2279957 | 0.010371 | 2.0883157 | down |
| Bivm | Mus musculus basic, immunoglobulin-like variable motif containing (Bivm), mRNA [NM_144558] | A_55_P2170109 | 0.011495 | 2.0465457 | up |
| Cyp2r1 | Mus musculus cytochrome P450, family 2, subfamily r, polypeptide 1 (Cyp2r1), mRNA [NM_177382] | A_55_P1966755 | 0.041499 | 2.40151 | down |
|  |  | A_55_P2095864 | 0.032325 | 2.19626 | down |
| Jag1 | Mus musculus jagged 1 (Jag1), mRNA [NM_013822] | A_52_P634090 | 0.04412 | 2.0774941 | down |
| Atp6v0d2 | Mus musculus ATPase, H+ transporting, lysosomal V0 subunit D2 (Atp6v0d2), mRNA [NM_175406] | A_66_P124179 | 0.025687 | 2.1549447 | up |
| Mfsd2a | Mus musculus major facilitator superfamily domain containing 2A (Mfsd2a), mRNA [NM_029662] | A_51_P279437 | 0.017035 | 2.1160913 | down |
| 4933411E02Rik | Mus musculus adult male testis cDNA, RIKEN full-length enriched library, clone:4933411E02 product:unclassifiable, full insert sequence. [AK016767] | A_55_P2305430 | 0.006394 | 2.6019063 | up |
|  |  | A_55_P1986095 | 0.036451 | 2.2897396 | up |
|  | predicted gene 9967 [Source:MGI Symbol;Acc:MGI:3704300] [ENSMUST00000068836] | A_55_P2044542 | 0.007056 | 2.50227 | up |
| Bhmt | Mus musculus betaine-homocysteine methyltransferase (Bhmt), mRNA [NM_016668] | A_55_P2105181 | 0.033883 | 4.2849984 | up |
| Agk | Mus musculus acylglycerol kinase (Agk), nuclear gene encoding mitochondrial protein, mRNA [NM_023538] | A_55_P2063163 | 0.005518 | 3.0436656 | up |
|  |  | A_55_P2083894 | 0.015471 | 2.6255677 | down |
| 1600020E01Rik | Mus musculus cDNA clone IMAGE:5027084. [BC029726] | A_55_P2356840 | 0.014341 | 2.8497224 | up |
| Tmtc3 | Mus musculus transmembrane and tetratricopeptide repeat containing 3 (Tmtc3), transcript variant 2, mRNA [NM_001033332] | A_51_P212473 | 0.005088 | 2.0404403 | up |
| Glo1 | Mus musculus glyoxalase 1 (Glo1), transcript variant 1, mRNA [NM_025374] | A_51_P480982 | 0.049039 | 2.0451524 | down |
| C78653 | PREDICTED: Mus musculus expressed sequence C78653 (C78653), misc_RNA [XR_105388] | A_55_P2301058 | 0.013319 | 3.0300176 | up |
| Lce1i | Mus musculus late cornified envelope 1I (Lce1i), mRNA [NM_029667] | A_51_P206475 | 0.024677 | 2.168835 | up |
| Camk2a | Mus musculus calcium/calmodulin-dependent protein kinase II alpha (Camk2a), transcript variant 1, mRNA [NM_009792] | A_55_P2149209 | 0.00371 | 2.0192168 | up |
| C130030J05 | Mus musculus cDNA clone IMAGE:6832152. [BC098190] | A_55_P2321468 | 0.02568 | 2.2166426 | up |
|  | predicted gene 10680 [Source:MGI Symbol;Acc:MGI:3704335] [ENSMUST00000156612] | A_55_P2136561 | 0.034451 | 2.0268943 | down |
| Cstf3 | Mus musculus cleavage stimulation factor, 3' pre-RNA, subunit 3 (Cstf3), transcript variant 1, mRNA [NM_145529] | A_51_P382928 | 0.010892 | 2.641156 | up |
|  | predicted gene 5146 [Source:MGI Symbol;Acc:MGI:3646305] [ENSMUST00000088673] | A_55_P2070676 | 0.024549 | 2.079544 | up |
|  |  | A_55_P2037787 | 0.006688 | 2.4940221 | down |
