# Supplementary material for: Gene Transcriptional and Metabolic Profile Changes in Mimetic Aging Mice Induced by D-Galactose
Source: PLoS One. 2015 Jul 15;10(7):e0132088. doi: 10.1371/journal.pone.0132088 (PMC4503422; doi:10.1371/journal.pone.0132088)
Supplement: S5 Table — (DOCX) [file pone.0132088.s005.docx]

S5-Table The results of total RNA extraction

| Sample ID | 浓度 (µg/µl) | A260/  A280 | A260/  A230 | 28S/18S | 2100RIN |
| --- | --- | --- | --- | --- | --- |
| D-galactose-1 | 0.3605 | 2.15 | 2.14 | 0.9 | 7.0 |
| D-galactose-2 | 0.3809 | 2.07 | 2.13 | 1.0 | 7.0 |
| D-galactose-3 | 0.3799 | 2.15 | 2.13 | 1.0 | 8.2 |
| control-1 | 0.2259 | 2.12 | 1.94 | 1.6 | 9.0 |
| control-2 | 0.3044 | 2.05 | 1.95 | 1.4 | 8.4 |
| control-3 | 0.5120 | 2.14 | 2.13 | 1.3 | 8.8 |

The values of A260/A280 are between 2.05 and 2.15, that means the purity of RNA can meet the experiment requirements. RNA concentration is between 0.2259 and 0.5120.

The values of 28S/18S and 2100RIN were greater than 0.7and 7，respectively. That means RNA have good integrity。
