# Supplementary material for: Gene Transcriptional and Metabolic Profile Changes in Mimetic Aging Mice Induced by D-Galactose
Source: PLoS One. 2015 Jul 15;10(7):e0132088. doi: 10.1371/journal.pone.0132088 (PMC4503422; doi:10.1371/journal.pone.0132088)
Supplement: S7 Table — (DOCX) [file pone.0132088.s007.docx]

**The technology roadmap of our experiment and the explanation of supporting information**

ICR mice were randomly divided into two groups

10 male ICR mice 10 male ICR mice

injected D-galactose at a dose of 120 mg/kg/day for 6 weeks

Treated with saline (0.9%) the same volume for 6 weeks

D-galactose aging group control group

the content of MDA, activities of CAT、SOD、GSH –Px in mouse liver and brain were determined. (**Supporting Information S1 Table and S2 Table**)

gene expression profiles of the two groups’ livers are studied using the Agilent Mouse cDNA genechip( **as shown in fig. 1**)

changes of metabolites of the two groups’ livers are studied using the GC/MS-based metabonomics approach(**S3 Table . Details of changes of metabolites detected by GC/MS**).

**fig. 1**

RNA extraction、quantification and quality control

( **S5-Table The results of total RNA extraction**)

cRNA synthesis、labeling、hybridization and washing

Microarray scanning and Raw data extraction

Raw data normalization

**(S6 Table. standardize data)**

Differential gene screening

**(S4 Table . Details of significant mRNA changes)**
